# Titrating Gene Function in the Human Fungal Pathogen Candida albicans through Poly-Adenosine Tract Insertion

**DOI:** 10.1128/mSphere.00192-19

**Published:** 2019-05-22

**Authors:** Helene Tournu, Arielle Butts, Glen E. Palmer

**Affiliations:** a Department of Hematology and Oncology, College of Medicine, University of Tennessee Health Sciences Center, Memphis, Tennessee, USA; b Department of Clinical Pharmacy and Translational Science, College of Pharmacy, University of Tennessee Health Sciences Center, Memphis, Tennessee, USA; Carnegie Mellon University

**Keywords:** *Candida*, poly-A, *albicans*, gene expression, gene function, protein expression, protein function, translational control

## Abstract

A recent study demonstrated that the insertion of poly-adenosine (poly-A) tracts into an open reading frame can suppress expression of the encoded protein in both prokaryotic and eukaryotic species. Furthermore, the degree of suppression is proportional to the length of the poly-A insertion, which can therefore provide a reliable and predictable means to titrate a specific protein’s expression. The goal of this study was to determine if this methodology can be applied to modulate the expression of proteins in the prevalent human fungal pathogen, Candida albicans. Insertion of increasing numbers of AAA codons encoding lysine at the N terminus of the C. albicans lanosterol demethylase (Erg11p) progressively diminished expression without significantly reducing the levels of mRNA. This suggests that Erg11p expression was attenuated at the posttranscriptional level. A direct correlation between the number of AAA codons inserted and C. albicans susceptibility to the Erg11p inhibitor fluconazole was also noted, indicating a progressive loss of Erg11p activity. Finally, we constructed a series of C. albicans strains with 3 to 12 AAA codons inserted at the 5′ end of the *ARO1* gene, which encodes a pentafunctional enzyme catalyzing five sequential steps of the aromatic amino acid biosynthetic pathway. Increasing numbers of AAA codons progressively reduced the growth rate of C. albicans in standard laboratory medium, indicating a progressive loss of ARO biosynthetic activity. These data unequivocally demonstrate the potential utility of the poly-A insertion method to examine the phenotypic consequences of titrating target protein function in C. albicans.

**IMPORTANCE** Investigating a protein’s functional importance at the whole-organism level usually involves altering its expression level or its specific activity and observing the consequences with respect to physiology or phenotype. Several approaches designed to partially or completely abolish the function of a gene, including its deletion from the genome and the use of systems that facilitate conditional expression, have been widely applied. However, each has significant limitations that are especially problematic in pathogenic microbes when it is desirable to determine if a particular gene is required for infection in an animal model. In this study, we sought to determine if an alternative approach—the insertion of poly-A repeats within the coding sequence of the gene—is sufficient to modulate its function in the prevalent human fungal pathogen C. albicans. Our results confirm that this approach enables us to predictably and gradually titrate the expression level of a protein and thus to investigate the phenotypic consequences of various levels of gene/protein function.

## Abstract (precis)

Investigating a protein’s functional importance at the whole-organism level usually involves altering its expression level or its specific activity and observing the consequences with respect to physiology or phenotype. Several approaches designed to partially or completely abolish the function of a gene, including its deletion from the genome and the use of systems that facilitate conditional expression, have been widely applied. However, each has significant limitations that are especially problematic in pathogenic microbes when it is desirable to determine if a particular gene is required for infection in an animal model. In this study, we sought to determine if an alternative approach—the insertion of poly-A repeats within the coding sequence of the gene—is sufficient to modulate its function in the prevalent human fungal pathogen C. albicans. Our results confirm that this approach enables us to predictably and gradually titrate the expression level of a protein and thus to investigate the phenotypic consequences of various levels of gene/protein function.

## INTRODUCTION

The functional importance of a protein at the whole-cell or whole-organism level is commonly determined by altering its expression level or biological activity and observing the consequences with respect to viability and/or physiology. Ideally, production of the protein of interest would be completely abolished through deletion of its coding sequence from the genome. However, this is not possible when a protein is essential for viability, as complete deletion of the coding sequence is lethal. Furthermore, it is often desirable to assess the consequences of diminished rather than complete loss of function. Several approaches have been devised that enable partial or conditional suppression of a protein’s expression or biological activity and can be applied to study essential genes. These include strategies that alter the steady-state level of an mRNA transcript by exchanging the endogenous promoter for an ectopic or conditional promoter to change the rate of transcription initiation. Other methods such as the use of RNA interference (RNAi) or antisense RNA or destabilizing the transcript through disruption of the 3′ untranscribed region (3′UTR) sequences of genes (decreased abundance by mRNA perturbation [DAmP]) reduce the half-life of mRNA by changing its rate of degradation (1, 2). A protein’s steady-state expression level can also be modulated by manipulating the efficiency of mRNA translation, i.e., the rate of translation initiation or peptide elongation. For example, the rate of translation can be profoundly impacted by the use of “preferred” or “unpreferred” codons (3). Additionally, according to the N-end rule, a protein’s half-life is determined by the amino acid exposed at its N terminus, and molecular processing strategies that facilitate its substitution have been applied to alter steady-state expression levels (4). Finally, point mutations that alter a protein’s amino acid composition can increase or decrease its stability or biological or catalytic activity or can confer conditional function in the form of, e.g., temperature-sensitive alleles. However, it is often difficult to predict the degree to which a particular protein’s expression or function may be impaired using any of these strategies. Moreover, it is rarely possible to produce a series of strains with a range of functionality using these methodologies. These approaches are even more restrictive in pathogenic microbes when it is desirable to establish a protein’s validity as a target for antimicrobial development using strains with altered target function in an animal model of infection. In such cases, it is not always possible to manipulate the host organism’s temperature or the availability of nutrients to control transcription from regulatable promoters.

Recently Arthur and colleagues (5, 6) described an alternative approach to modulate a specific protein’s expression level—the addition of poly-adenosine (poly-A) tracts within its coding sequence. The sequential addition of lysine-encoding AAA triplets progressively diminished protein expression levels in both prokaryotic and eukaryotic species. However, the addition of an equivalent number of AAG codons, also encoding lysine, did not affect the target protein’s expression. The addition of poly-A tracts is proposed to attenuate protein translation through two distinct mechanisms: (i) the synthesis of stretches of amino acids with a high density of positive charge can stall peptide elongation on the ribosome due to electrostatic interactions with the negatively charged peptide exit channel (7), and (ii) there is evidence that poly-A tracts can cause “ribosome slippage,” resulting in translational frameshifts and, consequently, mistranslation and/or premature peptide termination (8). Ultimately, these events result in degradation of both the aberrant nascent polypeptide chain and the mRNA transcript through established pathways (9), releasing and rescuing the bound ribosome. Thus, poly-A tracts may reduce the efficiency of a protein’s translation and decrease the longevity of the corresponding mRNA. The goal of this study was to determine if this methodology can be applied to titration of gene function in the prevalent human fungal pathogen, Candida albicans.

## RESULTS

To determine how the insertion of poly-A tracts into the coding sequence of a C. albicans gene affects its function, we focused on the following: (i) lanosterol demethylase (Erg11p), a key enzyme in the ergosterol biosynthetic pathway and the target of the azole antifungals (10), and (ii) Aro1p, a pentafunctional protein that catalyzes five sequential steps within the aromatic amino acid biosynthetic pathway (11) and is a prospective target for novel antifungal development (12).

### Inserting poly-A tracts into the *ERG11* coding sequence produced Candida albicans strains with a range of protein expression levels.

**FIG 1:**
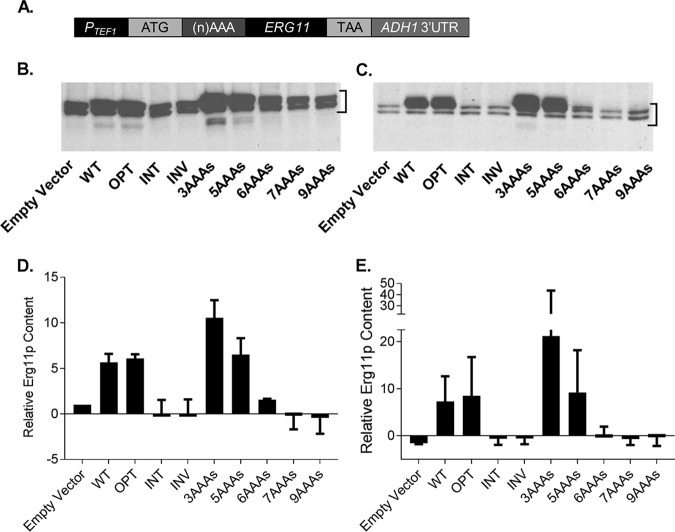
Insertion of polylysine-encoding tracts of increasing length progressively diminished Erg11p expression levels in Candida albicans. (A) Schematic representation of the Erg11p expression constructs used in these studies. The C. albicans *ERG11* coding sequence was amplified with 0, 3, 5, 6, 7, or 9 consecutive AAA codons introduced at the N terminus, and the products were then cloned into the pKE4 expression vector. In addition, three synthetic Erg11p coding sequences, *ERG11^OPT^*, *ERG11^INT^*, and *ERG11^INV^*, each with altered usage of synonymous codons, were produced and cloned into the same vector. Each construct or vector alone was then introduced into the *URA3* locus of a strain in which the transcription of the endogenous *ERG11* allele can be suppressed with doxycycline (*erg11Δ*/*TETO-ERG11*). (B and C) Each strain was grown in YPD medium (B) or in YPD plus 10 μg/ml doxycycline (C) for 5 h at 30°C; extracts were then prepared and equal amounts of protein analyzed by Western immunoblotting with a polyclonal antiserum against Erg11p. A representative blot is shown for each set of growth conditions. (D and E) Quantitation of Erg11p expression data from panels B (YPD) and C (YPD plus 10 μg/ml doxycycline), respectively. The signal intensity for Erg11p was measured using ImageJ software, and the results were normalized to total protein for each sample. The multiple bands shown on the blots likely represent Erg11p with and without heme (personal communications from Steve Kelly, University of Swansea). Data are expressed for each sample relative to that determined for the empty vector strain grown in YPD medium (D), which was designated the reference sample. Data represent means ± standard deviations of results from two biological samples.

**FIG 2:**
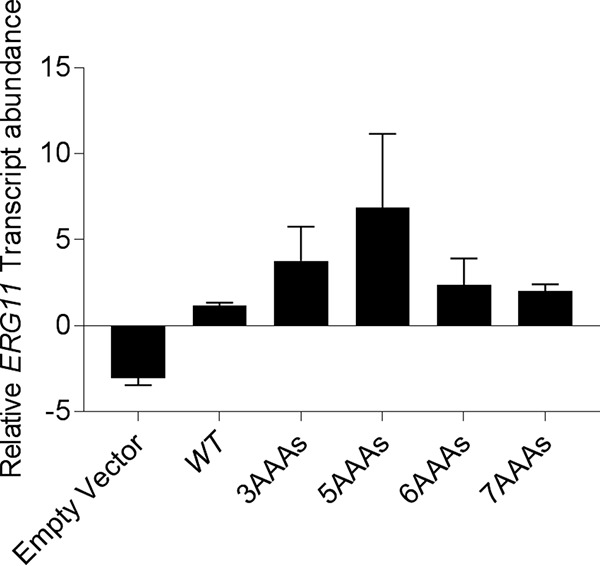
Insertion of polylysine-encoding tracts did not predictably affect the abundance of the *ERG11* transcript in Candida albicans. Strains harboring an *ERG11^WT^*, *ERG11^3AAA^*, *ERG11^5AAA^*, *ERG11^6AAA^*, or *ERG11^7AAA^* expression construct were grown in the presence of 10 μg/ml doxycycline for 6 h to suppress expression of the endogenous *ERG11* allele before RNA was extracted. The abundance of the *ERG11* transcript in each sample was then determined by qRT-PCR and normalized to that of the *ACT1* gene (loading control). Data presented represent means ± standard deviations of results from two biological samples.

To demonstrate that the insertion of poly-A sequences into the coding sequence of a C. albicans gene reduces the expression of the encoded protein, we amplified the *ERG11* open reading frame (ORF) using oligonucleotides that incorporated 3, 5, 6, 7, or 9 AAA codons immediately following the ATG start codon. Each product was cloned into a previously described expression vector, pKE4, to drive transcription from the powerful C. albicans *TEF1* promoter (*P_TEF1_*) (Fig. 1A). The resulting constructs were introduced into a strain in which one of the endogenous *ERG11* alleles was deleted and the second was placed under the control of a doxycycline-repressible promoter (*erg11Δ*/*P_TETO_-ERG11*) (13). Using this strain background enabled us to tightly repress expression of the endogenously encoded *ERG11* allele and therefore to investigate the relative expression levels and functions of the recombinant *ERG11* alleles. For comparison, we explored how changes in codon usage affected the expression and function of Erg11p. Three synthetic versions of the C. albicans *ERG11* coding sequence were produced with alternative uses of synonymous codons. These included a version in which codon usage was aligned to those preferred in genes that produce highly abundant proteins (*ERG11^OPT^*—optimized); a second version which was designed to use the least preferred codons associated with highly expressed proteins (*ERG11^INV^*—inversely optimized); and a third version that had suboptimal codon usage (*ERG11^INT^*—intermediate). All three versions, as well as the wild-type (WT) allele (*ERG11^WT^*), were cloned into the same expression vector and introduced into the *erg11Δ*/*P_TETO_-ERG11* strain background. We then compared the levels of Erg11p expression in the engineered strains both with and without (minus) suppression of transcription from the remaining endogenous allele with doxycycline. Previous studies performed with the *erg11Δ*/*P_TETO_-ERG11* strain have shown that Erg11p expression becomes limiting for growth approximately 6 h after exposure to doxycycline (13). Western blot analysis confirmed that Erg11p expression was suppressed by doxycycline at that time point relative to minus doxycycline control cultures (Fig. 1B to E). The introduction of the pKE4-*ERG11^WT^* expression construct into the *erg11Δ*/*P_TETO_-ERG11* strain elevated the level of expression of Erg11p approximately 5-fold to 6-fold in the absence of doxycycline versus the vector-alone control strain, indicating that the majority of Erg11p produced in these cells is expressed from the pKE4 construct, even in the absence of doxycycline. The *ERG11^OPT^* coding sequence yielded a level of Erg11p expression similar to that seen with the wild-type *ERG11* sequence in both the presence and absence of doxycycline, while both the *ERG11^INT^* and *ERG11^INV^* versions gave only very low levels of Erg11p expression, barely above that seen with the vector-alone control strain. Unexpectedly, the addition of 3 AAA codons to *ERG11* (*ERG11^3AAA^*) actually increased Erg11p expression compared to the level seen with *ERG11^WT^*. However, the sequential addition of AAA codons progressively depleted Erg11p expression levels, as expected (5, 6), such that the *ERG11^7AAA^* and *ERG11^9AAA^* strains were comparable in that respect to the vector-alone control strain. Consistent with the protein expression data, quantitative reverse transcription-PCR (qRT-PCR) revealed that the *ERG11* transcript was more abundant in the *ERG11^3AAA^* strain than in the *ERG11^WT^* strain (Fig. 2). However, the relative transcript abundances in the *ERG11^5AAA^*, *ERG11^6AAA^*, and *ERG11^7AAA^* strains did not correspond to the protein expression data, with the *ERG11* transcript most abundant in the *ERG11^5AAA^* strain and the *ERG11^6AAA^* and *ERG11^7AAA^* strains having levels similar to that seen with the *ERG11^WT^* control strain (Fig. 2). Thus, the primary mechanism responsible for attenuating Erg11p expression in the AAA repeat strains does not appear to involve reducing mRNA abundance. We cannot, however, completely rule out the possibility that poly-AAA insertions may have other effects on gene expression that can affect mRNA processing. Nevertheless, these data demonstrate that the insertion of poly-A tracts of increasing lengths into the coding sequence of C. albicans genes can be used to progressively suppress the expression of the encoded protein. In contrast, while changing codon usage profoundly impacted the expression of Erg11p, we were not able to demonstrate a predictable titration.

### Inserting poly-A tracts into the Candida albicans *ERG11* coding sequence facilitated phenotypic titration.

**FIG 3:**
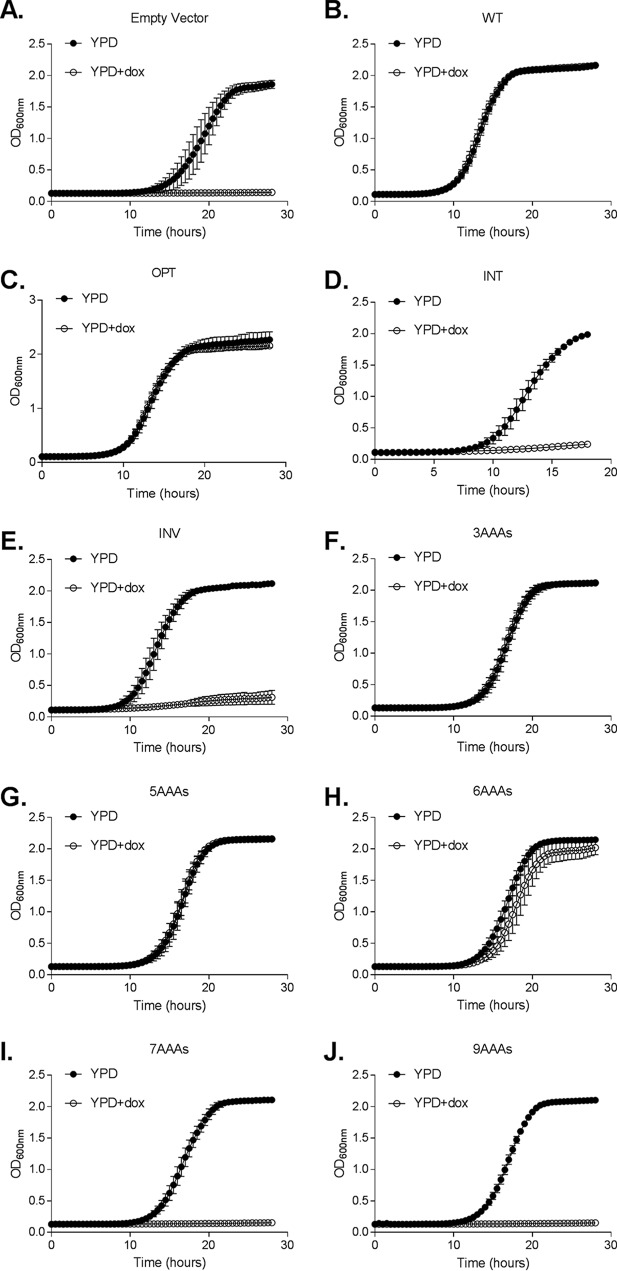
Insertion of polylysine-encoding tracts was sufficient to generate hypomorphic *ERG11* alleles in Candida albicans. C. albicans strains expressing each of the *ERG11* variants (panels A to J) were inoculated into YPD or YPD plus 10 μg/ml doxycycline at 5 × 10^3^ cells/ml and incubated at 30°C. Growth was then monitored by measuring OD_600_ at 30-min intervals. For each allelic variant, four independent transformants (and two technical repeats for each) were used to calculate the average growth rates and standard deviations.

We next examined the relationship between the length of the poly-A insertion and target gene function by assessing the growth rate of the engineered *ERG11* strains. As expected, suppression of *ERG11* transcription with doxycycline strongly suppressed growth of the vector-alone control strain (Fig. 3A), whereas the strains harboring either the pKE4-*ERG11^WT^* construct or the pKE4-*ERG11^OPT^* construct continued to grow (Fig. 3B and C). In contrast, neither the *ERG11^INT^* coding sequence nor the *ERG11^INV^* coding sequence was sufficient to support C. albicans growth following suppression of transcription of the endogenous allele (Fig. 3D and E). While the *ERG11^3AAA^* and *ERG11^5AAA^* alleles were able to sustain a normal rate of C. albicans growth (Fig. 3F and G), the strain harboring *ERG11^6AAA^* allele had a slightly reduced growth rate upon doxycycline treatment compared to the minus doxycycline control culture and the *ERG11^WT^* control strain (Fig. 3H), indicating that Erg11p function is limiting for growth. The strains expressing either *ERG11^7AAA^* or *ERG11^9AAA^* were completely unable to grow upon doxycycline-mediated suppression of the endogenous *ERG11* allele (Fig. 3I and J), indicating insufficient Erg11p activity to support C. albicans growth.

**FIG 4:**
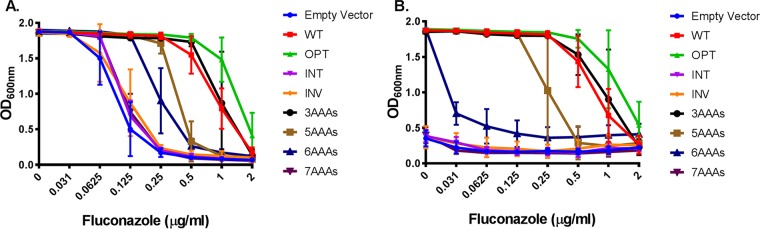
Insertion of polylysine-encoding tracts of increasing length into *ERG11* progressively increased Candida albicans susceptibility to fluconazole. C. albicans strains expressing each of the *ERG11* variants were seeded into either YNB medium with increasing concentrations of fluconazole (A) or YNB plus 10 μg/ml doxycycline with increasing concentrations of fluconazole (B) at 5 × 10^3^ cells/ml. Growth was then measured as the OD_600_ after 48 h of incubation at 30°C. The data presented for each allelic variant represent means ± standard deviations of results from four independently derived transformants with two technical replicates for each, i.e., a total of eight data points.

Finally, we examined Erg11p function in whole cells by determining the sensitivity of each strain to fluconazole, an antifungal drug that directly and selectively inhibits this enzyme. This revealed that, following doxycycline-mediated suppression of the *P_TETO_-ERG11* allele, growth of the *ERG11^WT^*-expressing strain was inhibited by ≥0.5 μg/ml fluconazole (Fig. 4). The sensitivity of the *ERG11^3AAA^*-expressing strain was similar to that of the *ERG11^WT^* control strain, while the *ERG11^5AAA^* strain was 2-fold to 4-fold more sensitive, suggesting that those strains had broadly similar levels of Erg11p activity. Consistent with a lower level of Erg11p activity, the *ERG11^6AAA^* strain was sensitive, with ≤0.031 μM fluconazole being sufficient to inhibit growth (∼16-fold increase in fluconazole sensitivity versus the *ERG11^WT^* control). As expected, the growth levels of the vector-alone control strain and the *ERG11^7AAA^*, *ERG11^INT^*, and *ERG11^INV^* strains were substantively inhibited in the presence of doxycycline alone.

### The insertion of AAA repeats into *ARO1* produced a panel of Candida albicans strains with increasingly severe deficiencies in aromatic amino acid biosynthesis.

Finally, we sought to demonstrate the potential utility of the poly-A insertion methodology to investigate the function of a less extensively characterized protein, Aro1p, a pentafunctional enzyme that catalyzes five sequential steps of the shikimate pathway, which is central to aromatic amino acid biosynthesis (14). The *ARO1* gene has been previously shown to be essential for C. albicans viability and pathogenicity in a mouse model of disseminated infection by the use of a doxycycline-repressible expression system (12, 15). However, we were able to demonstrate that *ARO1* is not an essential gene in C. albicans *in vitro*, as we were able to construct and propagate an *aro1Δ*/Δ mutant on medium containing high concentrations of tryptophan, phenylalanine, and tyrosine (10 mM each) as the sole nitrogen sources (TTP medium) (data not shown). Nonetheless, the deletion strain was completely unable to grow in any standard growth medium.

**FIG 5:**
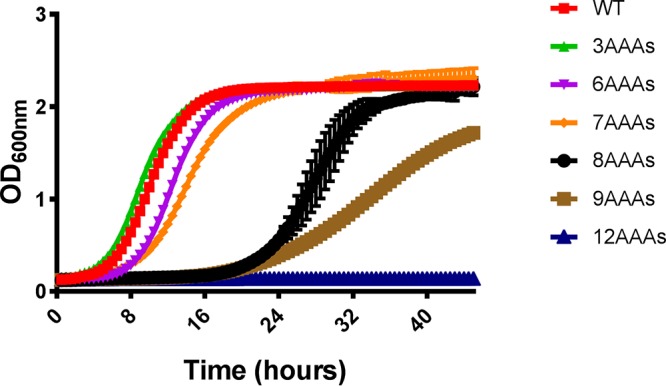
Insertion of polylysine-encoding tracts of increasing length into *ARO1* progressively suppressed Candida albicans growth. C. albicans strains expressing either a wild-type *ARO1* allele or an *ARO1^3AAA^*, *ARO1^6AAA^*, *ARO1^7AAA^*, *ARO1^8AAA^*, *ARO1^9AAA^*, or *ARO1^12AAA^* allele were seeded to YNB medium at 5 × 10^3^ cells/ml and incubated at 30°C. Growth was then monitored by measuring the OD_600_ at 30-min intervals. Means ± standard deviations of results from two independently derived isolates representing each genotype are shown.

A series of C. albicans strains were constructed in which one allele of *ARO1* was completely deleted and replaced by the *ARG4* selection marker and the promoter of the second allele was replaced with the *TEF1* promoter followed by an ATG start codon and up to 12 AAA codons, in frame with the remaining coding sequence. In the absence of an Aro1p-specific antibody, we used growth of the engineered strains in yeast extract-peptone-dextrose (YPD) medium at 30°C by measuring the optical density at 600 nm (OD_600_) at 30-min intervals as a proxy for Aro1p function. This revealed that while the insertion of 3 AAA codons had a minimal effect, if any, on the growth rate of C. albicans, the insertion of longer poly-A tracts resulted in progressively more severe growth defects (Fig. 5). The experiments performed with the *ARO1^6AAA^* and *ARO1^7AAA^* alleles resulted in a moderate but significant reduction in growth, while the *ARO1^8AAA^* and *ARO1^9AAA^* strains showed severe growth defects with a prolonged lag phase as well as a reduced rate of growth in the exponential phase. Lastly, the strain harboring the *ARO1^12AAA^* allele was completely unable to grow in YPD medium. These data unequivocally demonstrate the potential utility of the poly-A insertion method to examine the phenotypic consequences of titrating target gene function in C. albicans.

## DISCUSSION

To date, the most widely adopted strategies for investigating the function of essential C. albicans genes have utilized conditional expression systems that permit the suppression of target gene transcription. This typically involves deleting one allele from the genome and placing the second allele under the control of an exogenous promoter, the activity of which can be regulated (15–17). Phenotypic comparisons are then made under conditions of promoter induction and repression. For example, the *MET3* promoter, whose activity can be suppressed in medium containing high concentrations of cysteine and/or methionine, is commonly used for this purpose (17). The doxycycline-repressible expression system has also been widely used in C. albicans (15) and has been especially valuable for suppressing target gene expression in animal models of infection (12). While both have provided important insights, they also have significant limitations.

First, shifts in amino acid or carbon source availability or the addition of xenobiotics required to regulate the target gene’s transcription can have a profound impact on global gene expression patterns and the physiology of the yeast. This can make direct comparison of phenotypes under inducing versus repressing conditions problematic. Of significant concern, doxycycline is known to impair mitochondrial function in eukaryotic cells (18) and may therefore have unintended effects on the physiology of both the fungus and its mammalian host when used in animal models of C. albicans infection. Doxycycline is also likely to have a profound impact on the endogenous microbiota of mammalian species, which may in turn have a significant impact on host physiology and immune function. The degree to which these undesirable effects may influence outcomes of experiments using animal models of C. albicans infection is unknown. One of the major advantages of using poly-A-mediated repression of the target protein is that it does not require manipulation of growth conditions or the use of xenobiotics to suppress target protein expression and therefore is not prone to the aforementioned complications.

Second, approaches that rely upon exogenous factors to regulate expression of the target gene are often not well suited to investigation of the phenotypic consequences of target protein titration. The capacity to achieve a desirable range of target protein expression or activity depends upon several factors, including the stringency of the system’s regulation, its sensitivity and responsiveness to the regulator, levels of target protein expression occurring under basal as well as repressing/inducing conditions, and the relative abundance of the target protein expressed from the native promoter in unmanipulated cells, as well as the sensitivity of the cell to reduced target protein function. For example, the level of target gene transcription from such nonnative promoters under nonrepressing conditions can be several orders of magnitude higher or lower than the level seen with the endogenous promoter. As a result, the expression level of the gene of interest may be insufficient to sustain normal cell physiology. Alternatively, overexpression of the gene product may lead to mislocalization within the cell or to the sequestration of interacting components away from other cellular functions, potentially resulting in indirect phenotypic consequences. Expression from regulatable promoters may also be “leaky” under repressing conditions, allowing low levels of transcription that, for some genes, may be enough to sustain cell viability. Thus, the relative degrees of target protein expression and function that can be attained under both repressing and nonrepressing conditions can be difficult to predict and are likely to differ greatly between target genes, complicating the interpretation of phenotypic data. Consequently, the relative levels of effectiveness of regulatable expression systems in investigations of gene function are expected to differ greatly according to a number of target-specific factors.

Further restricting the utility of these systems, it is not possible to change the nutritional environment or alter other factors necessary to control conditional promoters within mammalian tissue, i.e., in animal models of infection. For example, even though gene expression can be titrated in C. albicans using the doxycycline-repressible system in a dose-dependent manner *in vitro* (15, 19), it is challenging in a practical sense to endeavor to achieve graded expression within an animal model. This is especially the case when doxycycline is provided in the animals’ food or water, as is commonly the case, due to wide variations in consumption by individual animals. The capacity to progressively diminish target protein function without the need to manipulate external factors, such as growth conditions or the use of xenobiotics, makes the poly-A method extremely attractive as a method to investigate target gene function, especially in animal models of infection. Moreover, comparing the levels of pathogenicity of a panel of strains with various levels of function of a specific protein is especially appealing for the purpose of drug target validation as it may reveal the sensitivity of the infecting microbe to interference with target protein function. This is important to model since many drugs inhibit target protein activity only partially under physiological conditions or at pharmacological concentrations. Data from experiments involving titration of target protein activity are particularly valuable for target engagement studies where it is crucial to establish a correlation between levels of target protein activity and the outcome of disease. This information can help establish a criterion for selecting potentially efficacious drug candidates on the basis of their *in vitro* activity profile and is crucial for interpretation of a drug’s performance at both the preclinical and clinical stages of development (20). Finally, while more-traditional gene replacement methods were used to introduce the poly-A insertions into the target genes in this study, the advent and widespread application of clustered regularly interspaced short palindromic repeat (CRISPR)-Cas9-based methods in *Candida* spp. as well as pathogenic fungi (21, 22) will make the poly-A insertion methods even more convenient.

As anticipated, completely changing the usage of synonymous codons throughout the whole coding sequence of *ERG11* had a profound impact on protein expression and function. However, in our experiments performed with the four alleles that were compared in this study, we did not observe a progressive titration of either Erg11p expression levels or biological function as the codon usage shifted from codons favored by to those disfavored by highly expressed genes. Instead, we observed an “all or nothing” response, with the “optimized” allele performing similarly to the wild-type coding sequence and the suboptimal coding sequence being nonfunctional. While the work described here did not rule out the possibility that changes in the usage of synonymous codons can be applied to titrate the expression of a specific protein, it did indicate that this approach may produce less-predictable effects than the poly-A method.

One potential complication of the method as applied in this study is the possibility of a target protein being completely intolerant of modifications, e.g., insertion of polylysine or other epitope tags at the selected site. For example, in attempting to apply the method to the nonessential C. albicans *ERG3* gene, we observed that insertion of 3 or more AAA codons at the 5′ end of the ORF resulted in a complete rather than gradual loss of function, as evidenced by results representing an *erg3* null mutant phenotype (see Fig. S1 in the supplemental material). This is consistent with other evidence indicating that C. albicans Erg3p is intolerant of any modifications at its N terminus (unpublished results). Nonetheless, previous evidence suggested that it should be possible to suppress target protein expression irrespective of the poly-A insertion site, e.g., insertions at the the N or C terminus or intrapeptide insertions (5, 6). Furthermore, a simple control method to determine the impact of polylysine insertion upon target protein function is to insert an equivalent number of AAG codons or another epitope tag in place of the AAA codons. Related to these issues is the mechanism by which the presence of poly-A tracts leads to decreased gene expression. Arthur and colleagues proposed previously that the principal mechanisms are those of reduced efficiency and fidelity of protein production (5, 6); however, it is also possible for any given target protein that the addition of multiple lysines may affect protein folding, biological activity, or half-life.

10.1128/mSphere.00192-19.1 FIG S1 Candida albicans Erg3p is intolerant of polylysine insertion at its N terminus. A C. albicans *ERG3*/*erg3Δ* strain was constructed, and the promoter of the second allele was replaced with the *TEF1* promoter sequence such that 0, 3, 6, or 9 AAA codons were introduced at the N terminus of the coding sequence. (A and B) *ERG3* function was then examined by comparing the rates of growth of the strains on YPD agar or YPD agar supplemented with SDS, CaCl_2_, or fluconazole using spot dilution assays (A), or rates of hyphal growth were compared on M199 agar (B). Wild-type (SC5314) and *erg3Δ*/Δ strains of C. albicans were used as a control. (C) RNA was extracted from the indicated strains and the abundance of the *ERG3* transcript determined by qRT-PCR. Data for each strain were then normalized to the data determined for the *ACT1* transcript. A minimum of three independent transformants for each strain transformed with a *TEF1* promoter construct and a minimum of two biological replicates for the wild-type and heterozygote strains were used to calculated the means and standard deviations. Download FIG S1, PDF file, 0.4 MB. Copyright © 2019 Tournu et al. 2019 Tournu et al. This content is distributed under the terms of the Creative Commons Attribution 4.0 International license.

Finally, we sought evidence of the presence of poly-A tracts within the coding sequence of C. albicans genes to determine whether this mechanism might influence the expression of endogenous proteins. We identified 328 C. albicans genes encoding three or more consecutive lysines, totaling just under 1,400 codons. Extracting the corresponding coding sequences of these lysine-repeat motifs from genome sequences revealed that 51% were AAG codons and 49% were AAA codons. In contrast, considering all of the lysine codons occurring within all predicted C. albicans open reading frames, the AAA codon (∼70%) was shown to be strongly preferred over the AAG codon (∼30%) (23). The AAA codon is hence highly underrepresented within polylysine-encoding tracts of C. albicans (χ^2^ = 431.2; df = 1; *P* < 0.0001), perhaps indicating the presence of selective pressure against poly-A sequences within the coding portions of the genome. Among those genes encoding polylysine tracts, 29 exclusively contained AAA codons, with up to 7 consecutive AAA codons found in *PUS1*, which encodes a putative tRNA pseudouridine synthase. Clustering of the biological processes and cellular components (http://funspec.med.utoronto.ca/) (24) of the Saccharomyces cerevisiae homologues of these 29 genes (when present) revealed overrepresentation of tRNA and rRNA modification processes and of the nucleolus, respectively (*P* < 0.001). The endogenous occurrence of long poly-AAA tracts is hence a rare phenomenon in C. albicans that seems to be highly specific to ribosomal assembly and processing.

## MATERIALS AND METHODS

### Growth conditions.

C. albicans was routinely grown on yeast extract-peptone-dextrose (YPD) agar plates at 30°C. Selection of C. albicans transformants was carried out on minimal yeast nitrogen base (YNB) medium (6.75 g liter^−1^ yeast nitrogen base without amino acids, 2% dextrose, 2% Bacto agar), supplemented with the appropriate auxotrophic requirements or 50 μg ml^−1^ uridine, unless otherwise stated. For RNA and protein extraction, cells were grown for 6 h in YPD medium unless otherwise stated. For the *ERG11*-related studies, doxycycline was used at a final concentration of 10 μg/ml. To generate the hypomorphic strains in the *aro1Δ*/*ARO1* background, cells were grown on minimal medium lacking ammonium sulfate and amino acids with 2% dextrose and supplemented with 10 mM tryptophan, tyrosine, and phenylalanine.

### Plasmid construction.

Plasmids pLUX (25), pGEMHIS1 and pRSARG4ΔSpe (26), and pKE4 containing a *TEF1* promoter and *ADH1* terminator (27) have been previously described. To facilitate replacement of endogenous transcriptional promoters in C. albicans, a previously described plasmid constructed in pGEMHIS1 with the *P_TEF1_* promoter sequence (565 bp) was utilized (28).

All oligonucleotides used in this study are listed in Table S1 in the supplemental material. For analysis of expression of the *ERG11* hypomorphic alleles, the *ERG11* ORF was subjected to PCR amplification from wild-type genomic DNA using forward primers, X-AAAs-ERG11-F-SalI (containing a poly-A tract of 0, 3, 5, 6, or 7 lysine codons), and ERG11ORFR-MluI and was digested with SalI and MluI for cloning between the same sites of pKE4 to produce pKE4-ERG11, pKE4-3AAAs-*ERG11*, pKE4-5AAAs-*ERG11*, pKE4-6AAAs-*ERG11*, and pKE4-7AAAs-*ERG11* plasmids.

10.1128/mSphere.00192-19.2 TABLE S1 List of primers used in this study. Download Table S1, DOCX file, 0.01 MB. Copyright © 2019 Tournu et al. 2019 Tournu et al. This content is distributed under the terms of the Creative Commons Attribution 4.0 International license.

### Candida albicans strains.

SC5314 (29) and BWP17 (26) have been previously described. C. albicans was transformed with DNA constructs using the lithium acetate procedure (30). All strains generated in this study were made prototrophic, and they are listed in Table S2. The heterozygote *ERG3*/*erg3Δ* and *erg3Δ*/Δ gene deletion strains were previously described (31). Hypomorphic alleles were introduced using the PCR-based approach (26). *ERG3*-targeting primers ERG3PRF and X-AAAs-ERG3PRR were used to amplify P*_TEF1_* using pGEM-P*_TEF1_*-*HIS1* as a template, hence containing up to 60 bases of homology with *ERG3* sequence upstream of the ATG start site and the poly-AAA tracts in the reverse primers. These PCR products were used to replace the *ERG3* promoter with the *TEF1* promoter and to introduce the poly-AAA tracts at the N-terminal end of *ERG3* in the heterozygote strain background. Correct insertion upstream of the *ERG3* ORF was then confirmed using TEF1prDETF and ERG3-DET-R primers.

10.1128/mSphere.00192-19.3 TABLE S2 List of strains used in this study. Download Table S2, DOCX file, 0.02 MB. Copyright © 2019 Tournu et al. 2019 Tournu et al. This content is distributed under the terms of the Creative Commons Attribution 4.0 International license.

The *erg11Δ*/*P_TETO_-ERG11* strain was previously described (13). The hypomorphic alleles of *ERG11* were introduced into *ura3^−^* recipient *erg11Δ*/*P_TETO_-ERG11* following digestion of pKE4 empty vector, pKE4-*ERG11*, and pKE4-*ERG11^XAAA^* with NheI to target integration into (and to reconstitute) the *URA3* loci (32). Correct integration and thus reconstitution of the *URA3* loci were confirmed by PCR with primers LUXINTDETF/R.

The coding sequence of *ERG11* was optimized according to the codon bias of a subset of highly expressed, ribosomal C. albicans proteins using the OPTIMIZER program (33). Sequences for the optimized version (codon adaptation index [CAI] value of 1), the inversely optimized version (CAI of 0.087), and an intermediately optimized version (CAI of 0.193) of *ERG11* were generated using one AA-one codon, one inverted AA-one codon, and inverted guided random settings, respectively. Synthetic sequences incorporating SalI and MluI sites on either side of each optimized coding sequence were produced by IDTDNA, amplified from the supplied DNA template using primers AMPF1 and AMPR1, and cloned between the SalI and MluI sites of the pKE4 vector.

To generate the *ARO1*/*aro1Δ* strain background, the *ARO1* deletion gene deletion cassette was amplified using ARO1DISF and ARO1DISR with pRSARG4ΔSpeI (*ARG4* selection marker) as the template and transformed into BWP17. Correct integration of the gene deletion cassette was confirmed by diagnostic PCR using primers ARG4INTR2 and ARO1-DIA-F as well as ARG4INTF2 and ARO1-DIA-R to confirm replacement of one *ARO1* allele with the *ARG4* selection marker. The *ARO1* replacement cassettes were amplified using ARO1PRF and ARO1PRR primers with the pGEMHIS1-based promoter plasmid described above as the template. Correct insertion of the desired promoter upstream of the *ARO1* ORF was then confirmed using ARO1-DIA-R2 and TEF1prDETF. The hypomorphic alleles were constructed as follows. Poly-A tracts of the desired length were introduced in the X-AAAs-ARO1-PRR primers, and, together with the ARO1-PRF primer, were used to amplify P*_TEF1_* from pGEM-*P_TEF1_*-*HIS1*. Correct integration was confirmed by PCR as described above.

### Fluconazole susceptibility assays.

Antifungal susceptibility testing was performed in 96-well plates using the broth microdilution method described in the M27-A3 CLSI guidelines, with the following modifications. Assays were performed in unbuffered minimal medium to ensure that doxycycline retained its activity. Fluconazole (Sigma-Aldrich) was diluted in dimethyl sulfoxide (DMSO) using 2-fold dilutions at 200 times the final concentration, hence resulting in a final DMSO concentration of 0.5%. The cell inoculum was 1 × 10^3^ cells per well. Plates were incubated at 30°C for 24 and 48 h. Growth was measured at OD_600_ using a Cytation 5 cell imaging multimode reader (Bio-Tek Instruments, Inc.).

### Time course growth assays.

Cultures were prepared at 5 × 10^4^ cells/ml and divided into aliquots of 200 μl in 96-well plates. Growth curves were generated using a Cytation 5 cell imaging multimode reader (Bio-Tek Instruments, Inc.) and a continuous-shaking setup at 30°C. Absorbance was measured at 600 nm every 30 min for the indicated periods of time. Averages and standard deviations were calculated from at least two independent transformants for each strain background.

### RNA extraction.

Cells were collected at 3,500 rpm for 3 min and immediately frozen at −80°C after removal of the supernatant. RNAs were extracted using the hot phenol method as previously described (34). RNA pellets were eluted in 20 μl of nuclease-free water. RNA quantity and purity were determined by measuring absorbance at 260 nm and 280 nm.

### Quantitative RT-PCR.

Aliquots of 1 μg/ml RNA were treated with DNase I as indicated by the manufacturer (Thermo Scientific). Random hexamers were used to synthesize cDNA by the use of a Verso cDNA synthesis kit (Thermo Scientific) according to the manufacturer’s instructions. Quantitative PCR was performed using Maxima SYBR green/ROX quantitative master mix (Thermo Scientific) with primer pairs ACT1FWDS2 and ACT1REVS2 to amplify *ACT1*, ERG3qPCR_F2 and ERG3qPCR_R2 to amplify *ERG3*, and ERG11-1345F and ERG11-1430R to amplify *ERG11*. Samples were processed in a model 7500 real-time PCR system (Applied Biosystems). *ACT1* was used to normalize the data, and expression levels of each target gene were calculated using the threshold cycle (ΔΔ*C_T_*) method as previously described (35). Experiments were performed with two to four independent biological replicate cultures in technical triplicate. Statistical analysis was performed using the Student's *t* test.

### Immunoblot analyses.

C. albicans strains were grown overnight in YPD medium at 30°C and subcultured to an OD_600_ of 0.2 in 10 ml of fresh YPD medium. Doxycycline was added at a final concentration of 10 μg/ml. All cultures were grown for 5 h at 30°C and collected via centrifugation. Total protein extracts were prepared as previously described (28). In brief, cell pellets were resuspended in 200 μl lysis buffer (50 mM Tris-HCl [pH 7.5], 150 mM NaCl, 1 mM EDTA, 1% Triton X-100) with added protease inhibitor cocktail (Roche) and 0.5-mm-diameter glass beads. Samples were lysed by 10 cycles of 20-s bursts with a bead beater followed by 60 s on ice. The total protein concentration was determined using a Bradford assay (Thermo Scientific) dye concentrate according to the manufacturer's instructions. A 50-μg volume of total protein of each sample was fractionated on a 10% SDS-PAGE Mini-Protean TGX gel (Bio-Rad), transferred to a nitrocellulose membrane, and blocked with 5% nonfat milk–TBST buffer (50 mM Tris [pH 7.5], 150 mM NaCl, 0.05% Tween 20). The membranes were then probed with an anti-Erg11 antibody (kindly provided by Steve Kelly, Swansea University) (36) followed by anti-rabbit horseradish peroxidase (HRP)-conjugated secondary antibody (Bio-Rad). HRP conjugate was detected using a Clarity ECL Western blotting detection system (Bio-Rad). Blots were imaged using a G:Box Chemi XT-4 system (Syngene) and analyzed using ImageJ (NIH).
