# Supplementary figures and images for: Titrating Gene Function in the Human Fungal Pathogen Candida albicans through Poly-Adenosine Tract Insertion
Source: mSphere. 2019 May 22;4(3):e00192-19. doi: 10.1128/mSphere.00192-19 (PMC6531883; doi:10.1128/mSphere.00192-19)

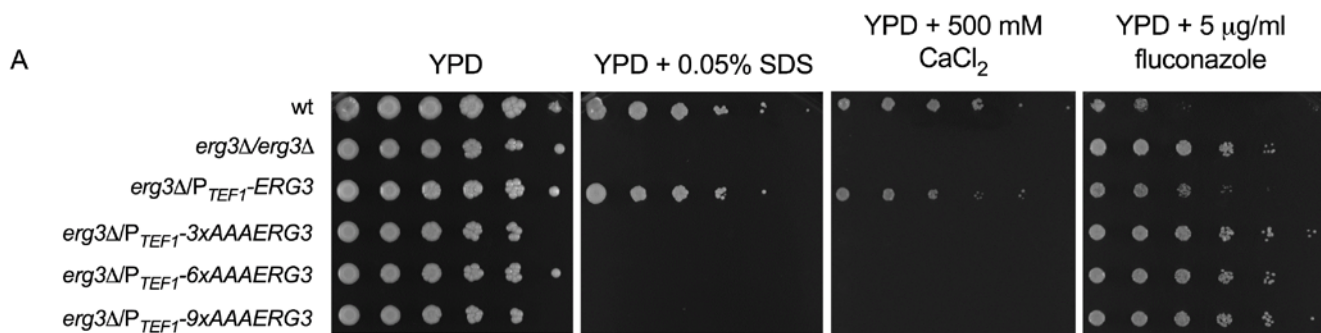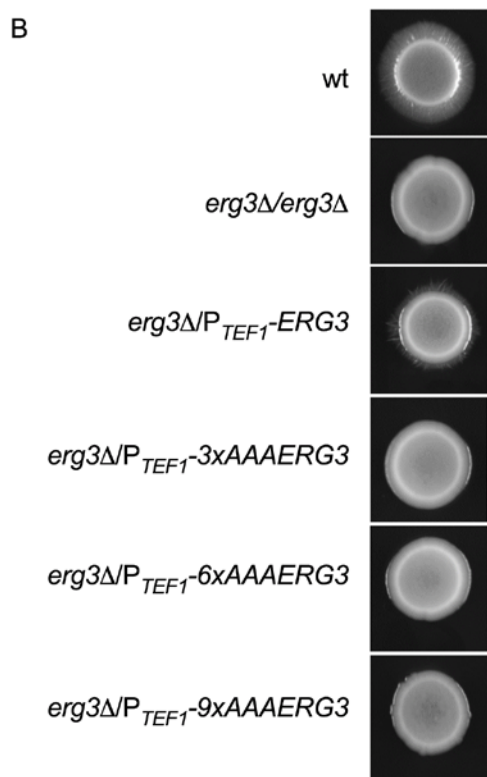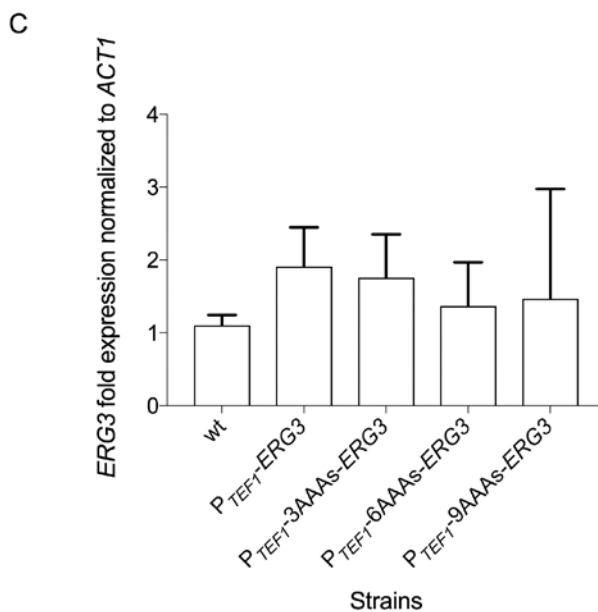

Supplement: FIG S1 [file mSphere.00192-19-sf001.pdf]
