# Supplementary material for: Titrating Gene Function in the Human Fungal Pathogen Candida albicans through Poly-Adenosine Tract Insertion
Source: mSphere. 2019 May 22;4(3):e00192-19. doi: 10.1128/mSphere.00192-19 (PMC6531883; doi:10.1128/mSphere.00192-19)
Supplement: TABLE S1 [file mSphere.00192-19-st001.docx]

**Supplemental table 1. Oligonucleotides used in this study.**

| ***Primer*** | ***Sequence*** ***5’→3’**** |
| --- | --- |
| ERG11ORFR-MluI | TCAACGCGTTGAATCGAAAGAAAGTTGCCG |
| ERG11ORFF-SalI | TCAGTCGACATGGCTATTGTTGAAACTGTC |
| 3AAAs-ERG11ORFF-SalI | GCGAAGTCGACATGAAAAAAAAAGCTATTGTTGAAACTGTCATTGATGGCATTAATTATTTTTTGTCCCTTAGTGTTACACAA |
| 5AAAs-ERG11ORFF-SalI | GCGAAGTCGACATGAAAAAAAAAAAAAAAGCTATTGTTGAAACTGTCATTGATGGCATTAATTATTTTTTGTCCCTTAGTGTTACACAA |
| 6AAAs-ERG11ORFF-SalI | GCGAAGTCGACATGAAAAAAAAAAAAAAAAAAGCTATTGTTGAAACTGTCATTGATGGCATTAATTATTTTTTGTCCCTTAGTGTTACACAA |
| 7AAAs-ERG11ORFF-SalI | GCGAAGTCGACATGAAAAAAAAAAAAAAAAAAAAAGCTATTGTTGAAACTGTCATTGATGGCATTAATTATTTTTTGTCCCTTAGTGTTACACAA |
| ARO1DISF | ATGTCTATTGAAAAGGTGCCAATTTTGGGTAAGGAAACTATCCATGTTGGTTATGGTATTGCCGACCATATGTGGAATTGTGAGCGGATA |
| ARO1DISR | TCAACAACAGCACGATGAATAATCTCATATGGTGCAGTAAAACCAGTATGAAGTTTAAACTGTCTATCACGTTTTCCCAGTCACGACGTT |
| ARO1DIAF | ATGGCTCATCGTGCTGCCA |
| ARO1DIAR | ACGTTGATATCCAATCGC |
| ARG4INTF2 | AAGCTAGTGTGGAAAGAAGAG |
| ARG4INTR2 | AATGACTGAATTATGTCGGTC |
| ARO1PRF | CATAAGAAAGACTTCCTCCAGTATGTCTTAGTTTTTACCCTTGTTGGTCATGGCTCATCGTGCTGAGTTGAACTCCCTTATGGTGC |
| ARO1PRR | TCGGCAATACCATAACCAACATGGATAGTTTCCTTACCCAAAATTGGCACCTTTTCAATAGACATACGTCGCGTGCTCCCGGCCG |
| 3AAAs-ARO1PRR | TCGGCAATACCATAACCAACATGGATAGTTTCCTTACCCAAAATTGGCACCTTTTCAATAGATTTTTTTTTCATACGTCGCGTGCTCCCGGCCG |
| 6AAAs-ARO1PRR | TCGGCAATACCATAACCAACATGGATAGTTTCCTTACCCAAAATTGGCACCTTTTCAATAGATTTTTTTTTTTTTTTTTTCATACGTCGCGTGCTCCCGGCCG |
| 7AAAs-ARO1PRR | TCGGCAATACCATAACCAACATGGATAGTTTCCTTACCCAAAATTGGCACCTTTTCAATAGATTTTTTTTTTTTTTTTTTTTTCATACGTCGCGTGCTCCCGGCCG |
| 8AAAs-ARO1PRR | TCGGCAATACCATAACCAACATGGATAGTTTCCTTACCCAAAATTGGCACCTTTTCAATAGATTTTTTTTTTTTTTTTTTTTTTTTCATACGTCGCGTGCTCCCGGCCG |
| 9AAAs-ARO1PRR | TCGGCAATACCATAACCAACATGGATAGTTTCCTTACCCAAAATTGGCACCTTTTCAATAGATTTTTTTTTTTTTTTTTTTTTTTTTTTCATACGTCGCGTGCTCCCGGCCG |
| 12AAAs-ARO1PRR | TCGGCAATACCATAACCAACATGGATAGTTTCCTTACCCAAAATTGGCACCTTTTCAATAGATTTTTTTTTTTTTTTTTTTTTTTTTTTTTTTTTTTTCATACGTCGCGTGCTCCCGGCCG |
| ERG3PRF | TCAACTAAGGTCAACCTTCCCATCACATTACTGCTTACTTTGAGAGGTTCTTTAACAGTTTCCCAAGTTGAACTCCCTTATGGTGC |
| ERG3PRR | GGGAAAACATCAGCATAAACTTTATCAAAAAGATAATAGTCACAAATTTCTAGTACGATATCCATACGTCGCGTGCTCCCGGCCG |
| 3AAAs-ERG3PRR | GGGAAAACATCAGCATAAACTTTATCAAAAAGATAATAGTCACAAATTTCTAGTACGATATCTTTTTTTTTCATACGTCGCGTGCTCCCGGCCG |
| 6AAAs-ERG3PRR | GGGAAAACATCAGCATAAACTTTATCAAAAAGATAATAGTCACAAATTTCTAGTACGATATCTTTTTTTTTTTTTTTTTTCATACGTCGCGTGCTCCCGGCCG |
| 9AAAs-ERG3PRR | GGGAAAACATCAGCATAAACTTTATCAAAAAGATAATAGTCACAAATTTCTAGTACGATATCTTTTTTTTTTTTTTTTTTTTTTTTTTTCATACGTCGCGTGCTCCCGGCCG |
| ARO1-DIA-R2 | CTTGAACAACTCTGACACCTC |
| TEF1prDETF | ATGGATCAGCTGTGCTTGTGG |
| LUXINTDETF | CTGACCTTTAGTCTTTCCTGC |
| LUXINTDETR | CAGTAGTACTTGTTGTTGTATCG |
| ERG3-DET-R | GTAAGAGTTACCAAGTCTATCCC |
| ACT1FWDS2 | ACTACCATGTTCCCAGGTATTG |
| ACT1REVS2 | CCACCAATCCAGACAGAGTATT |
| ERG11-1345F | CTGATGAAGTTGATTATGGG |
| ERG11-1430R | ATCTATGTCTACCACCACCA |
| ERG3qPCR_F2 | CTGGTCTAATGACCCAGTTGTC |
| ERG3qPCR_R2 | CCAAGTCTATCCCAAAGAGTAGTG |

*Restriction sites are underlined
