# Supplementary material for: Titrating Gene Function in the Human Fungal Pathogen Candida albicans through Poly-Adenosine Tract Insertion
Source: mSphere. 2019 May 22;4(3):e00192-19. doi: 10.1128/mSphere.00192-19 (PMC6531883; doi:10.1128/mSphere.00192-19)
Supplement: TABLE S2 [file mSphere.00192-19-st002.docx]

**Supplemental table 2. List of strains used in this study.**

| **Strain** | **Genotype** | **Reference** |
| --- | --- | --- |
| SC5314 | Wild type | [1] |
| *erg11Δ*/P*_TETO_*-*ERG11* | *ura3*Δ/Δ:URA3 *his1*Δ/Δ *arg4* Δ/Δ *erg11*Δ:*ARG4*/ P*_TETO_*-*ERG11*:*HIS1* | [2] |
| *ERG1*1^WT^ | *ura3*Δ/Δ:*URA3*:*ERG11*^WT^ *his1*Δ/Δ *arg4* Δ/Δ *erg11*Δ:*ARG4*/ P*_TETO_*-*ERG11*:*HIS1* | This study |
| *ERG11*^OPT^ | *ura3*Δ/Δ:*URA3*:*ERG11*^OPT^ *his1*Δ/Δ *arg4* Δ/Δ *erg11*Δ:*ARG4*/ P*_TETO_*-*ERG11*:*HIS1* | This study |
| *ERG11*^INV^ | *ura3*Δ/Δ:*URA3*:*ERG11*^INV^ *his1*Δ/Δ *arg4* Δ/Δ *erg11*Δ:*ARG4*/ P*_TETO_*-*ERG11*:*HIS1* | This study |
| *ERG11*^INT^ | *ura3*Δ/Δ:*URA3*:*ERG11*^INT^ *his1*Δ/Δ *arg4* Δ/Δ *erg11*Δ:*ARG4*/ P*_TETO_*-*ERG11*:*HIS1* | This study |
| *ERG11*^3AAA^ | *ura3*Δ/Δ:*URA3*:*ERG11*^3AAA^ *his1*Δ/Δ *arg4* Δ/Δ *erg11*Δ:*ARG4*/ P*_TETO_*-*ERG11*:*HIS1* | This study |
| *ERG11*^5AAA^ | *ura3*Δ/Δ:*URA3*:*ERG11*^5AAA^ *his1*Δ/Δ *arg4* Δ/Δ *erg11*Δ:*ARG4*/ P*_TETO_*-*ERG11*:*HIS1* | This study |
| *ERG11*^6AAA^ | *ura3*Δ/Δ:*URA3*:*ERG11*^6AAA^ *his1*Δ/Δ *arg4* Δ/Δ *erg11*Δ:*ARG4*/ P*_TETO_*-*ERG11*:*HIS1* | This study |
| *ERG11*^7AAA^ | *ura3*Δ/Δ:*URA3*:*ERG11*^7AAA^ *his1*Δ/Δ *arg4* Δ/Δ *erg11*Δ:*ARG4*/ P*_TETO_*-*ERG11*:*HIS1* | This study |
| *ERG11*^9AAA^ | *ura3*Δ/Δ:*URA3*:*ERG11*^9AAA^ *his1*Δ/Δ *arg4* Δ/Δ *erg11*Δ:*ARG4*/ P*_TETO_*-*ERG11*:*HIS1* | This study |
| BWP17 | *ura3*Δ/Δ *his1*Δ/Δ *arg4* Δ/Δ | [3] |
| *ARO1/ aro1Δ* | *ura3*Δ/Δ:URA3 *his1*Δ/Δ:HIS1 *arg4* Δ/Δ *aro1*Δ*:ARG4/ARO1* | This Study |
| *ARO1*^WT^ | *ura3*Δ/Δ:URA3 *his1*Δ/Δ *arg4* Δ/Δ *aro1*Δ*:ARG4*/*HIS1*:P*_TEF1_*-*ARO1* | This study |
| *ARO1*^3AAA^ | *ura3*Δ/Δ:URA3 *his1*Δ/Δ *arg4* Δ/Δ *aro1*Δ*:ARG4*/*HIS1*:P*_TEF1_*-3AAA-*ARO1* | This study |
| *ARO1*^6AAA^ | *ura3*Δ/Δ:URA3 *his1*Δ/Δ *arg4* Δ/Δ *aro1*Δ*:ARG4*/*HIS1*:P*_TEF1_*-6AAA-*ARO1* | This study |
| *ARO1*^7AAA^ | *ura3*Δ/Δ:URA3 *his1*Δ/Δ *arg4* Δ/Δ *aro1*Δ*:ARG4*/*HIS1*:P*_TEF1_*-7AAA-*ARO1* | This study |
| *ARO1*^8AAA^ | *ura3*Δ/Δ:URA3 *his1*Δ/Δ *arg4* Δ/Δ *aro1*Δ*:ARG4*/*HIS1*:P*_TEF1_*-8AAA-*ARO1* | This study |
| *ARO1*^9AAA^ | *ura3*Δ/Δ:URA3 *his1*Δ/Δ *arg4* Δ/Δ *aro1*Δ*:ARG4*/*HIS1*:P*_TEF1_*-9AAA-*ARO1* | This study |
| *ARO1*^12AAA^ | *ura3*Δ/Δ:URA3 *his1*Δ/Δ *arg4* Δ/Δ *aro1*Δ*:ARG4*/*HIS1*:P*_TEF1_*-12AAA-*ARO1* | This study |
| *ERG3/erg3Δ* | *ura3*Δ/Δ:URA3 *his1*Δ/Δ:HIS1 *arg4* Δ/Δ *erg3*Δ*:ARG4/ERG3* | [4] |
| *erg3Δ/Δ* | *ura3*Δ/Δ:URA3 *his1*Δ/Δ *arg4* Δ/Δ *erg3*Δ*:ARG4/ erg3*Δ*:HIS1* | [4] |
| *ERG3^WT^* | *ura3*Δ/Δ:URA3 *his1*Δ/Δ *arg4* Δ/Δ *erg31*Δ*:ARG4*/*HIS1*:P*_TEF1_*-*ERG3* | This study |
| *ERG3*^3AAA^ | *ura3*Δ/Δ:URA3 *his1*Δ/Δ *arg4* Δ/Δ *erg31*Δ*:ARG4*/*HIS1*:P*_TEF1_*-3AAA-*ERG3* | This study |
| *ERG3*^6AAA^ | *ura3*Δ/Δ:URA3 *his1*Δ/Δ *arg4* Δ/Δ *erg31*Δ*:ARG4*/*HIS1*:P*_TEF1_*-6AAA-*ERG3* | This study |
| *ERG3*^9AAA^ | *ura3*Δ/Δ:URA3 *his1*Δ/Δ *arg4* Δ/Δ *erg31*Δ*:ARG4*/*HIS1*:P*_TEF1_*-9AAA-*ERG3* | This study |

**References**

[1] Tournu H, Luna-Tapia A, Peters BM, Palmer GE. 2017. In Vivo Indicators of Cytoplasmic, Vacuolar, and Extracellular pH Using pHluorin2 in Candida albicans. mSphere 2:e00276-17.

[2] Luna-Tapia A, Kerns ME, Eberle KE, Jursic BS, Palmer GE. 2015. Trafficking through the late endosome significantly impacts Candida albicans tolerance of the azole antifungals. Antimicrob Agents Chemother 59:2410-20.

[3] Robinson MD, Grigull J, Mohammad N, Hughes TR. 2002. FunSpec: a web-based cluster interpreter for yeast. BMC Bioinformatics 3:35.

[4] Gillum AM, Tsay EY, Kirsch DR. 1984. Isolation of the *Candida albicans* gene for orotidine-5′-phosphate decarboxylase by complementation of *S. cerevisiae ura3* and *E. coli pyrF* mutations. Molecular and General Genetics 198:179-182
